# Modulation of gut microbiota in healthy rats after exposure to nutritional supplements

**DOI:** 10.1080/19490976.2020.1779002

**Published:** 2020-08-26

**Authors:** Mirna Čoklo, Dina Rešetar Maslov, Sandra Kraljević Pavelić

**Affiliations:** Department of Biotechnology, Centre for High-throughput Technologies, University of Rijeka, Rijeka, Croatia

**Keywords:** Gut microbiota, microbiome, microbiome sequencing, proteomics methods, rat model, nutritional supplements

## Abstract

**Introduction:**

Rats are experimental animals, frequently used as model organisms in the biomedical studies, and increasingly used to study the gut microbiota. Specifically, the aim of latter studies is either the elucidation of relationship between intestinal dysbiosis and diseases or the determination of nutrients or pharmaceutical agents which can cause the modulation in the presence or abundance of gut microbiota.

**Aim:**

Herein, the research studies conducted on the gut microbiota of healthy rats are presented in a summarized and concise overview. The focus is on studies aimed to reveal the shifts in microbial composition and functional changes after exposure to various types of nutritional supplements.

**Methods:**

We performed the search of PubMed database using the term “rat gut microbiome microbiota” and examined studies aimed to assess the composition of gut microbiota in physiological homeostasis as well as the effect of various nutritional supplements on the gut microbiota of healthy rats.

## Introduction

In recent years microbiota studies increased in number and relevance, and different study models have been employed to better elucidate the occurrence and role of certain microbiota in health and disease. This subject is complex and researchers in the field might benefit from comprehensively presented data available for specific study models, such as for the animal models. The presented review, therefore, brings a systematic overview of the literature covering the topic of microbiota study in rat models, used widely in different *in vivo* studies. Rats are indeed, often used as model organisms to study the gut microbiota, to elucidate the relationship between intestinal dysbiosis and diseases and/or to analyze the effects of pharmaceutical agents or food supplements/nutrients on the microbiota status. The latter studies strongly rely on data and knowledge of the microbiota status in physiological conditions. The presented review summarizes relevant information through the sections covering specific subtopics. First, the general role of gut microbiota in mammals’ health is explained along with data on the formation and dynamics of the rat gut microbiota in the early life cycle. Second, the available rat models for the study of microbiota dysbiosis are presented, followed by the description of the physiology of microbiota in the gastrointestinal tract and feces of healthy rats. A paragraph explaining the use of rat models for human microbiota research in comparison with the mouse models is given, followed by two sections explaining known data on modulation of the gut microbiota in healthy rats by use of food supplements.

### Gut microbiota role in mammals’ health

It is assumed that the number of microbial cells in human microbiota is ten times greater (10^14^) than the total number of human cells (10^13^).^1^ Moreover, the gut microbiota in mammals is an extremely complex ecosystem, ranging from bacteria, viruses, and archaea to unicellular eukaryotes, such as fungi and yeast.^1^ Besides diversity of microbial composition, fluctuations in microbial relative abundance, and the variety of secreted functional molecules, microbial metabolites, also play an important role in the host health status.^2^ Due to such complexity and significance, the gut microbiota is often referred to as “the forgotten organ” or “the second genome”.^3,4^

Firstly, gut microbiota secrete enzymes, crucial for digestion of complex carbohydrates, such as resistant starches, plant cell wall polysaccharides, and non-digestible oligosaccharides.^5^ Secondly, gut microbiota perform vitamin synthesis, *i.e*. synthesis of cobalamin (vitamin B12), which is synthesized exclusively by anaerobic gut microorganisms. In addition, gut bacteria take part in the synthesis of vitamin K, biotin, folate, nicotinic acid, pantothenic acid, pyridoxine, riboflavin, and thiamine.^6^ Thirdly, the gut microbiota is essential for development and function of the host immune system. Indeed, bacterial colonization of the gut is crucial for normal development of the immunity, which was proven in the studies conducted on germ-free animals.^7^ Precisely, commensal and mutualistic bacteria protect the host against pathogenic species by: (1) competing for the same nutrients; (2) forming microenvironment unsuitable for the growth of parasitic species and (3) producing antimicrobial peptides or promoting T and B cell responses.^8^ Moreover, it was shown that the intestinal angiogenesis is also regulated by gut microbiota, for instance, the study by Stappenbeck *et al*. showed an arrested capillary network formation in adult germ-free mice. The latter state was successfully restarted and capillary formation was completed 10 d after the transplantation of gut microbiota was performed, by use of conventionally raised mice as donors or after the inoculation of single culture *Bacteroides thetaiotaomicron*.^9^ Finally, the gut microbiota is part of a complex communication system known as “gut-brain axis”. Microbiota interacts locally with enterocytes and the enteric nervous system, which also has a direct influence on the central nervous system through neuroendocrine and metabolic pathways, modulating behavior, motivation, and higher cognitive functions.^10^

It can be concluded that gut microbiota directly and indirectly influences the host health status through secreted functional molecules (proteins, peptides, and the molecules of low molecular weight) and influence pathogens as well.

As in humans, the formation of intestinal microbiota in rats occurs during and after birth, where neonatal rats are more exposed to fecal and environmental bacteria than humans.^11^ According to Yajima *et al*., during the first few weeks after birth, Gram-negative *Escherichia Coli* and Gram-positive *Lactobacillus* and *Streptococcus genus* dominate in the rat gut, while the anaerobic bacteria, *Bacterioidaceae*, and facultative anaerobic or microaerophilic *Lactobacilli* take over after weaning.^12^ Inoue and Ushida reported a clear change in the diversification of rat intestinal microbiota from suckling to maturity.^11^ Precisely, the first observed changes occur at 21–22 d after birth, and are due to weaning, diet change, and the simultaneous decrease in the maternal IgA levels. The second wave of changes occurs from d 24 to 27 after birth, probably attributed to the morphological and the immunological maturation of the gut.^12^ After the formative period is finished, the delicate equilibrium of gut microbiota is continuously perturbed by diet and environmental factors.

### Rat models for studies of microbiota dysbiosis

A link between gut dysbiosis and certain human diseases has been established so far, pointing to gut microbiota as an important topic in preventive medicine. Dysbiosis is often defined as an “imbalance” in the gut microbial community that is associated with disease. This imbalance could be due to the gain or loss of community members as well as changes in relative abundance of microbes.^13^ Dysbiosis or a definitive change of the normal gut microbiota with a breakdown of host-microbial mutualism is probably the defining event in the development of inflammatory bowel diseases.^14^ Also, changes in the gut microbiota are associated with specific metabolic states, such as obesity, diabetes, and metabolic syndrome.^15–17^ For instance, low fecal bacterial diversity is associated with marked overall adiposity and obese individuals have a higher abundance of *Firmicutes*, and nearly 90% lower abundance of *Bacteroidetes* in comparison with lean subjects.^18^ Changes in gut microbiota likely precede food allergies as well.^19^ Neuropsychiatric conditions, including autism, Parkinson’s disease, and depression are also states accompanied by changes in the gut microbiota.^20^ Recently, the topic of microbiota role in bone health, such as in osteoporosis has also been discussed in the scientific literature. The latter is based on the knowledge that microbiota has an effect on the bone.^21,22^ Acknowledging the importance of microbiota in some of the major medical issues of the modern world, studies are performed with the aim to establish scientifically based evidence on the correlation of the microbiota status with specific pathological states. However, this is an extremely complex research topic that requires a broad interdisciplinary and sophisticated methodological approach. Also, enormous complexity and a huge number of factors influencing the microbiota status in *real tim*e should be taken into account while performing such studies.

Animal models are accepted as an important research tool as they can be used to reduce and control parameters influencing the fluctuations and changes of the microbiota. In particular, rat models are a valuable tool for determining intestinal dysbiosis and the previously discussed human diseases relationship. Moreover, these models may help in discovery of nutrients or pharmaceutical agents which can prevent or reduce the microbiota alterations and gut microbiota dysbiosis.

The dextran sodium sulfate (DSS) colitis murine model has advantages over other various chemically induced experimental models due to its simplicity, reproducibility, and controllability. It may be particularly useful in the research of inflammatory bowel disease (IBD).^23^ Furthermore, according to Ghattamaneni *et al*. chronic administration of 0.5% DSS produces selective and reversible gastrointestinal changes in Wistar rats; increase of *Firmicute*s and decrease of *Bacteroidetes* and *Actinobacteria*, providing an improved chronic model in rats.^24^ Furthermore, metabolic syndrome as a combination of disorders that increases the risk of diabetes and cardiovascular diseases may be induced experimentally in rats fed with a fructose-rich diet.^25,26^ Previously, Srinivasan *et al*. determined that the combination of high-fructose diet and low-dose injections of streptozotocin in rats can serve as an alternative animal model for type 2 diabetes, simulating the human metabolic syndrome also suitable for testing anti-diabetic agents.^27^

One of the best rodent models for the study of autism and autism spectrum disorder (ASD) is the valproic acid-induced rat model. Using the latter experimental animal model, Liu *et al*. proved that valproic acid stimulates alterations in the microbiota features seen in autism, in addition to behavioral and anatomical changes characteristic for autistic brain.^28^ Rodent models, including rats, are extensively used in the discovery of novel treatments for Parkinson’s disease. Particularly, reserpine- and haloperidol-treated rats, 6-hydroxydopamine, and less frequently, rotenone and paraquat models, have proven as very useful.^29^ While some of the symptoms of depression are found exclusively in humans (guilt, suicidality, and sad mood), part of the depression symptoms can be replicated in laboratory rats (measures of helplessness, anhedonia, behavioral despair and other neurovegetative changes such as sleep alterations and appetite patterns) and moderated with antidepressant treatment.^30^ Differences in the gut microbiota composition between the depressive rat models and control animals were found, once more emphasizing the importance of gut-brain axis.^31^ The microbiota of depressed animals have similarities with those of depressive patients; for example, the richness of *Bacteroidetes* increases with a concomitant decrease of *Firmicutes* and abundance of *Lactobacillus*.^31^ In addition, Yu *et al*. found that relative abundances of the bacterial genera *Marvinbryantia, Corynebacterium, Psychrobacter, Christensenella, Lactobacillus, Peptostreptococcaceae incertae sedis, Anaerovorax, Clostridiales incertae sedis*, and *Coprococcus* were significantly decreased, whereas *Candidatus Arthromitus* and *Oscillibacter* were markedly increased in rats with chronic variable stress (CVS)-induced depression, compared with normal controls.^32^ Recently, different depression rat models were used as well, such as the olfactory bulbectomized rat, maternal separation, chronic variable stress-induced depression, and chronic restraint stress.^31^

### Physiology of microbiota in the gastrointestinal tract and feces of healthy rats

An important baseline for study of microbiota changes and dysbiosis is knowledge and information on the physiology of microbiota in the gastrointestinal tract and feces of healthy animals. This is why a number of studies were focused on the investigation of the microbial composition in the gastrointestinal tract and feces of healthy rats. Data from these studies represent a first baseline for microbiota research in rat models as well. Particularly, the contribution of recent studies is in comprehensive characterization of the so-called “normal” rat microbiota, which provides a basis for understanding and predicting disease-related alterations.^33^

The fecal flora of BioBreeding rats was, for example, analyzed by Brooks and coworkers by the use of two methods, namely (1) the randomly cloned 16 S rDNA comparative sequence analysis and (2) the bacterial cultures in different anaerobic media.^34^ The culture-independent approach provided deeper insights; however, only 20% of bacterial species, which were estimated to be present, were also successfully identified. For instance, the most dominant species of Gram-positive bacteria were *Lactobacilli*, representing 7% of in total 69 operational taxonomic units (OTUs). In addition, 16 S rDNA clones aligned with the *Clostridium coccoides* group (9%), the *Clostridium leptum* subgroup (18%), and Gram-negative *Bacteroides–Cytophaga* phylum. However, the majority of clone sequences were aligned with previously cultured, but still unknown bacterial species.^34^

Subsequently, a long-term consequence of cecal microbiota transplantation from Sprague-Dawley and Wistar rat strains on the intestinal microbiota of recipients Lewis strain rats was assessed by analyzing fecal samples in several rat model systems.^35^ In the control Lewis rat strain the authors identified 926 phylotypes with dominant phyla *Firmicutes* at 74% and *Bacteroidetes* at 23%. Obtained data allowed examining how different the rat and human intestinal microbiota are. The number of species in a fecal sample of control rat was two to three times higher than in fecal samples of two healthy human individuals. Finally, Manichanh *et al*. concluded that, at the phylum level, rat and human microbiota are similar, while specificity can be observed at the genus level.

In a detailed study, Li *et al*. performed the characterization of microbiota and microbial metabolites along the longitudinal axis of rat gastrointestinal (GI) tract, including feces.^33^ Results unambiguously revealed that the microbial biogeography of six male, pathogen-free Sprague-Dawley rats, which were held on a chow diet, is distinct from other murine animals, such as mouse or woodrat. Furthermore, the species richness and phylogenetic diversity increased from the upper to the lower GI segments, while the samples extracted from the colon mucus layer were of the highest richness and diversity. In mice, gastric, duodenal, and large-intestinal samples show similar diversity levels.^33^ Moreover, at the phylum level, 21 taxonomic groups were identified, but only *Bacteroidetes, Firmicutes, Proteobacteria*, and *Actinobacteria* were identified in all parts of the GI tract. Inter-individual microbiota variability is much higher in humans than in rats, and the authors attribute this to the similarity in genetic composition of laboratory rats, the uniform diet, the controlled environmental factors, and the coprophagy. Finally, in the gastrointestinal tract of healthy rats, the lactate-producing bacteria, such as *Lactobacillus* and *Turicibacter*, were dominant in the stomach and small intestine. In contrast, the core microbiota of the large intestine were anaerobic *Lachnospiraceae* and *Ruminococcaceae*.^33^

Furthermore, Flemer *et al*. showed that gut microbiota profiles may separate rats into three different clusters according to their age (1) before weaning, (2) first year of life (12- to 26-week-old animals) and (3) second year of life (52- to 104-week-old).^36^ A core of 46 bacterial species was present in all rats but relative abundance decreased progressively with age. This was accompanied by an increase of microbiota α-diversity (or number of different species in a sample), likely due to the acquisition of environmental microorganisms during the lifespan. In a study by Ferrario *et al*., the effect of three different dietary fibers on rat fecal microbiota was examined.^37^ A basal rat fecal microbiota content at the end of acclimatization week showed that *Bacteroidete*s (53.9%) represent the dominant phylum, outnumbering the *Firmicutes* (39.8%) and *Proteobacteria* (4%) phyla.^37^ *Actinobacteria*, unclassified members of *Saccharibacteria* phylum, *Cyanobacteria*, and *Tenericutes* together represent about 2% of the microbiota, while *Verrucomicrobia, Spirochaetae, Fusobacteria*, and *Elusimicrobia* phyla were determined at a low-level presence (≤0.1%).

As part of a study conducted by Nagpal *et al*., fecal microbiota composition of widely used animal models mice, rats, and non-human primates (NHPs) was analyzed using data generated on a single platform and with the same protocols.^38^ Data acquired was, subsequently, compared with those obtained for female (18 samples) and male (7 samples) human subjects confirming higher inter-individual variation in human gut microbiota.^38^ This comprehensive study revealed a more complex community of microbes present in rat feces, when compared to above-discussed data. In this study, the dominant phyla were *Bacteroidetes, Firmicutes* and *Proteobacteria*, followed by the *Spirochetes, Verrucomicrobia, Tenericutes* and hardly detectible *Actinobacteria*.^38^ In addition, relative abundance at the family level showed that the rat microbiota profile is distinct from other evaluated subjects, with the *Prevotellaceae* as the most dominant family. Also, *Bacterioidaceae, Clostridiales, Ruminococcaceae, Helicobacteraceae, Paraprevotellaceae*, and other less abundant families were detected.^38^ Species-specific unique bacterial profiles were also presented at the class- and order-level. However, the general patterns of gut microbiota abundance species were similar^38^ and the most abundant genera are presented in Table 1. When comparing the results with Li et al. (Table 1), we cannot make a straightforward conclusion. Since Li *et al*. found *Lactobacillus* and *Turicibacter* genera most abundant in the rat’s gastrointestinal tract, while it was not the case in rat feces from the study of Nagpal *et al*., it could be concluded that they confirm the results of Li *et al*. and that indeed fecal samples cannot represent the whole microbiota in the gastrointestinal tract. However, since rats in the study of Nagpal *et al*. were fed low fat and high-fat diets we cannot rule out the possibility that the differences in the results are not attributed to differences in rat’s diets as well as the influence of other confounding factors like rat’s age or housing environment. In line with the findings of the study conducted by Brooks *et al.*,^34^ the *Lactobacillus* genus was confirmed as the most abundant in rat feces. However, the same findings were not confirmed in mice, non-human primates, and human samples. The latter study, therefore, demonstrated that the microbiota profile of the rat is distinguishable from other evaluated species. Moreover, the authors showed that the microbiota profile of humans is more similar to that of non-human primates, when compared to rodents. On the other hand, the mice microbiota profile is more similar to human than to rat.^38^

**Table 1.** Microbiota of the rat gastrointestinal tract according to Li *et al*.^33^ and Nagpal *et al*.^38.^

Core microbiota of specific intestinal region or feces	Taxa (according to references^33,38^)	
	Li *et al.*	Nagpal *et al.*
Gastric content	*Turicibacter* (54.45%) Other taxa (28.08%) *Lactobacillus* (13.59%)	
Small-intestinal content	*Lactobacillus* (58.72%) Other taxa (15.55%) *Turicibacter* (13.65%) *Oscillibacter* (5.40%)	
Large-intestinal lumen	Other taxa (36.38%) *Lactobacillus* (25.08%) *Turicibacter* (17.24%)	
Mucus layer	Other taxa (38.87%) *Helicobacter* (12.36%) *Lactobacillus* (9.15%) *Turicibacter* (6.53%) *Flavonifractor* (5.50%) *Pseudoflavonifractor* (4.69%)	
Feces	Lactobacillus* (24.5%) Turicibacter* (22.72%) Taxa< 1% ave. abundance* (11.35%) unclassified bacteria* (belonging to *Porphyromonadaceae, Bacteroidetes* phylum) (5.5%)	*Prevotella* (29.4%) S24-7 genus (14.3%) *Clostridiales* genus (13.1%) *Helicobacter* (6.6%) *Ruminococcaceae* (4.4%) *Oscilospira* (4.0%) *Paraprevotellaceae* genus (3.4%) *Lactobacillus* (2.7%) *Bacterioides* (2.6%) *Treponema* (2.5%) *Ruminococcus* (2.3%) *Paraprevotella* (2.1%) *Rikenellaceae* genus (2%) *Clostridiaceae* genus SMB53 (1.9%) *Lachnospiraceae* (1.2%)

*The average relative abundance of dominant taxonomic groups at the genus level are given.

A first catalog of microbial genes in fecal samples of Sprague-Dawley (SD) rat was established recently.^39^ The study included analyses of 98 fecal samples, sampled at two time points, from 49 SD rats divided into 7 experimental groups. Intervention was the application of probiotic supplementation (*Lactobacillus casei*), methotrexate, and two Chinese experimental herb formulas to adjuvant-induced arthritis rat model. From 64.6% genes that were annotated to the phylum level, most of them belonged to *Firmicutes* (75.9%), *Bacteroidetes* (10.83%), and *Proteobacteria* (6.77%). From 26.7% genes that were annotated to the genus level most of them belonged to *Clostridium* (8.74%), *Bacteroides* (6.25%), *Roseburia* (4.75%), *Ruminococcus* (4.44%), and *Lachnoclostridium* (2.58%).^39^

### Rat models in human microbiota research and their comparison with mouse models

According to Hugenholtz and de Vos, rodent models used in human microbiota research, enable a rather easy collection of many samples from different sites of the gastrointestinal tract, allow multiple comparisons at a large scale, and offer a wide range of different genotypic backgrounds.^40^ A variety of information can be obtained from rodent models due to shared anatomical, histological, and physiological features of the gastrointestinal tract. At the same time, we have to keep in mind the differences, for instance, the morphological differences or dietary habits. Therefore, Hillman *et al*. provided an anatomical comparison of the gastrointestinal tract in humans and animal models,^41^ presented in Table 2.

**Table 2.** Comparison of the anatomy of the rat, mice, and human intestinal tract.^40–42.^

Part of the intestine	Organism		
	Rat	Mouse	Human
**Stomach**	Three regions: forestomach, body, and pylorus pH 3.0 to 4.0	Three regions: forestomach, body, and pylorus pH 3.0 to 4.0	Four regions: cardia, fundus, body, and pylorus pH 1.5 to 3.5
**Small intestine**	1485 mm in length pH 5.0 to 6.1	350 mm in length pH 4.7 to 5.2	5500–6400 mm in length pH 6.4 to 7.3
**Large intestine**	260 mm in length	140 mm in length	1500 mm in length
**Cecum**	Larger than the colon Main fermentation pH 5.9 to 6.6	Larger than the colon Main fermentation pH 4.4 to 4.6	Smaller than the colon No fermentation pH 5.7
**Appendix**	Absent	Absent	Present
**Colon**	Not divided No fermentation Thinner mucosa pH 5.5 to 6.2	Not divided No fermentation Thinner mucosa pH 4.4 to 5.0	Divided into the ascending, transcending, and descending colon Main fermentation Thick mucosa pH 6.7

Vdoviaková *et al*. presented the morphology of the stomach and intestine of adult Wistar rats of both genders and gave a comparison with the human gastrointestinal tract.^42^ The authors found that the anatomy of the rat stomach is greatly influenced by adaptation, nature of food, body size, and shape.^42^ Morphologically, they describe rat stomach as semilunar shaped sac weighing 1.8% of the total body weight while in humans stomach is pear-shaped sac weighing 6.2% of the total body weight.^42^ More importantly, unlike in humans, the rat stomach is divided into the forestomach (*pars proventricularis*) and glandular stomach (*corpus or pars glandularis*) comprising fundus and pylorus, with forestomach occupying about three-fifths of the stomach area and functionally serving as a storage organ.^42^ Another difference that the authors point out, is that humans have a poorly defined cecum, which is only continuous with the colon while rat cecum is as large as rat stomach.^42^ Also, colon in humans consists of the ascending, transverse, descending, and sigmoid sections with all parts of colon in human being sacculated on the other hand, the rat colon is simple and not sacculated.^42^ Regarding the dietary habits of laboratory rodents, Nguyen *et al*. noted that mice are fed with standardized chow diet throughout the experiment, which is composed mainly of plant materials and thus differs considerably to the usual composition and variation in a human daily diet.^43^ The same can be applied to rats. Nagpal *et al*. point out that mice and rats are herbivores with present coprophagy, while humans can be herbivores, carnivores, and omnivores based on their ethnicity, geography culture, and traditions.^38^

According to Franklin and Ericsson, rats are better suited for studies of microbiota as they provide a biological system similar to mice that is, however, large enough to better accommodate certain experimental techniques, *i.e*. colonoscopy and surgical manipulation.^44^ In addition, rats possess certain physiological parameters more closely related to those of humans.^44^ Moreover, Fritz *et al*. provided a comparison of the advantages and disadvantages of different animal models, including rat and mouse, commonly used for studying host–microbe interactions.^45^ The advantages of the rat models according to Fritz *et al*., are the availability of a number of rat-specific disease models or genetically altered rats with the completely sequenced genome. Also, they are relatively small in size and can be maintained easily.^45^ Further on, their reproduction is rather quick so that several generations can be observed in a relatively short period of time as they generally live 2 to 3 y.^45^ The disadvantage that Fritz *et al*. emphasized is expectedly, a diet and a living environment that differs substantially from those of humans.^45^ Mouse models, according to Fritz *et al*., have basically the same advantages as rat models, while disadvantages include again a living environment that differs substantially from those of humans and marked differences in the immune system and microbiota composition from those observed in humans.^45^

Generally, the rat, mouse, and human intestinal microbiota are similar at the phylum level but different at the genus level.^38,45^ The rat dominant phyla are *Firmicutes* (74%) and *Bacteroidetes* (23%).^45^ In humans, the dominant phyla are again the *Firmicutes* and *Bacteroidetes* as approximately 90% of bacterial species in the adult are members of these two phyla.^46^ Human microbiota also includes *Actinobacteria, Proteobacteria, and Verrucomicrobia* at the phylum level, and at lower proportions, *Fusobacteria, Tenericutes, Spirochetes, Cyanobacteria*, and TM7.^41^ At the lower levels of taxonomic classification, microbiome compositions vary with each individual.^41^ The mouse intestinal bacterial composition is also dominated by *Firmicutes* and *Bacteroidetes* phyla.^43^ According to Nguyen *et al*. genera with higher abundance in human gut microbiota in comparison with the mouse gut microbiota, include *Prevotella, Faecalibacterium*, and *Ruminococcus*, while *Lactobacillus, Alistipes*, and *Turicibacter* are more abundant in the mouse gut microbiota.^43^ In the study of Pan *et al*. where a catalog of microbial genes in fecal samples of Sprague-Dawley (SD) rat was established, the authors compared the obtained catalog with those of mouse and integrated human gut microbial gene catalogs, and found that only a low percentage of genes were shared by all three species, 1.29% in the rat, 0.58% in the human, and 2.72% in the mouse gut microbiota.^39^ They concluded that a comparison of the rat gut metagenome catalog with a human or a mouse revealed a higher pairwise overlap between rats and humans (2.47%) than between mouse and humans (1.19%).^39^ Additionally, Pan *et al*. noted that the potential of rats for biomedical research high because 97% of the functional pathways in the human catalog were present in the rat catalog as well.^39^ In the previously mentioned study by Nagpal *et al*., in which fecal microbiota composition of mice, rats, and non-human primates was compared to human subjects, the results showed that the gut microbiota, based on β-diversity (measure of diversity between communities) in humans seems to be closer to NHPs than to mice and rats, while mice microbiota appears to be closer to humans than rats.^38^ In the rat samples from Nagpal *et al*. study, genera represented with the highest frequency were *Prevotella* (29.4%), S24-7 (14.3%) and *Clostridiales* (13.1%), in mouse samples these were S24-7 (44.7%), *Clostridiales* (25.3%) and *Oscillospira* (5.0%), while in the human samples these were *Bacterioides* (27.5%), *Ruminococcaceae* (10.2%), and *Clostridiales* (9.7%).^38^ Additionally, according to Nguyen *et al*. who assessed the capability of mouse models to recapitulate the gut microbiota shifts associated with human diseases, rats are proposed to be more representative of the human gut microbiota than mice because the gut bacterial communities of humanized rats (germ-free rats as recipients of a human microbial community) reflect more closely the gut microbiota of human donors.^43^

Taking into account all the above-mentioned differences, rat models may be considered as a useful tool in the microbiome research due to the minimization of confounding experimental factors such as genetics, age, environment, and diet, which are all controlled in laboratory conditions. Nguyen *et al*. recognized clear differences which were observed at the level of specific genus/species abundances between the mouse and human gut microbiota, but still considered that although absolute comparison might be difficult, these models are relevant for studying microbiota variation and shifts upon disturbance.^43^ Mice are indeed, frequently used for evaluation of modulatory effect of different types of diets and nutrients on gut intestinal microbiota composition.^47–51^ We consider that the same relevance for studying microbiota shifts upon disturbance applies for rat models and present herein data on gut microbiota shifts in healthy rats after exposure to nutritional supplements. The observations of microbiota shifts in rats may be used as a ground for design of similar human microbiota research as well.

## Gut microbiota shifts in healthy rat models by different nutritional supplements

An interesting field of research are studies on the gut microbiota alterations in healthy rat models after dietary interventions. For this purpose, rat strains whose properties are presented in Table 3, such as Sprague-Dawley, Wistar, and Fischer 344 (F-334) rats, are the most commonly used, while Lewis, wild-type Groningen, and BioBreeding rats are not that commonly employed in these studies. As presented in Table 4, animals used within the same study are usually of the same sex, while male animals are more frequently utilized. The gender of animals, along with species, genetics, age, and factors such as diet, antimicrobials, and microenvironment, should be considered as potential confounding variables in the microbiota modulation studies.^11,87,88^ As dealing with confounding variables often relies on matching,^89^ scientists usually choose animals of the same gender in the experimental design. When effects of the gender on the microbiota composition were evaluated, the results proved inconsistent. Indeed, the gender as a variable has not been investigated in details as other factors, both in humans and in animals.^90^ A study by Org *et al*. showed that dietary effects on the composition and diversity of gut microbiota are partially dependent on sex-specific interactions. The authors examined sex differences related to the gut microbiota composition in a population of 89 common inbred mouse strains.^91^ Another study that showed the influence of gender on the microbiota composition was a study by Bernbom *et al*. where fecal suspension from a 32-y-old woman was administered to male and female GF rats. The afterward collected microbiota clustered according to the gender of the host animal.^87^ These findings should be taken into account in sex-comparative studies aimed to investigate potential health effects of diet as, for example, emphasized by Shastri *et al*. In their study administration of oligofructose increased the abundance of *Bacteroidetes* in female BioBreeding rats, but did not affect microbiota composition in males.^92^ At last, 1–2 months old rats are mainly used in experimental set-ups even though some studies rely on older rats as well (6-month-old).

**Table 3.** Properties of different rat strains.

Rat strain	Properties	Reference
Sprague-Dawley	Widely used outbred rat in biomedical research Good reproductive performance Genetically heterogeneous outbred rodents Albino strain of rats Ease in handling	52
Wistar	The outbred Wistar and Wistar HAN strains are used widely in Europe for preclinical safety assessments Wistar Kyoto and Wistar Furth rats are inbred strains A multipurpose model, *i.e*., infectious disease research, safety and efficacy testing, fracture models, and aging	53–56
Fischer 344 (F-334)	Previously used in toxicity and carcinogenicity studies In some models, F344 rat carcinogenicity studies lack relevance in predicting human carcinogenicity	57
Lewis (LEW)	Studies rewarding/reinforcing properties of drugs of abuse Impulsive traits Vulnerability to neuroinflammatory disease	58
wild-type Groningen	Studies on aggression and aspects of impulsivity Less vulnerable to social stress during adolescence in comparison to Wistar rats	59,60
BioBreeding rats	Diabetes-prone bio-breeding (DP-BB) rats spontaneously develop type 1 diabetes mellitus	61

**Table 4.** Overview of the studies of modulation of rat gut microbiota by different nutritional supplements.

Assessed nutritional supplement	Rats/experimental groups/sampling	Duration of study	Microbiota analyses – method overview	Key effects on gut microbiota	Reference
**Fruits, vegetables, nuts, pulses, cereal grains**					
**Lentil (*Lens culinaris Medikus*)**	**36, 8-week-old male Sprague-Dawley rats** **n = 12, standard diet** **n = 12, 3.5% high amylose corn starch diet** **n = 12, 70.8% red lentil diet** **Fecal samples collected and stored at −80°C.**	6 weeks	DNA extraction using QIAamp DNA Stool Minikit PCR Ilumina sequencing Microbial analysis-QIIME	↑ *Actinobacteria* (*Bifidobacterium spp*.) and *Bacteroidetes* ↓ *Firmicutes* ↓ *Lachnospiraceae spp*. ↓ *Clostridiales* order, *Peptostreptococcus spp., Lachnoanaerobaculum spp*.	^62^
**Walnuts**	**20, male Fischer 344 rats (mature, ˃250 g)** **n = 10, walnut** **n = 10, replacement** **Fecal samples collected from descending colon and stored at −80°C**	10 weeks	DNA extraction using QIAamp DNA Stool Kits PCR Illumina MiSeq sequencing platform Microbial analysis-QIIME	↑ species diversity ↑ *Firmicutes* ↓ *Actinobacteria, Cyanobacteria* ↑ *Oscillospira*, *Lachnospiraceae*, and *Turicibacter* ↑ *Lactobacillus, Ruminococcaceae* and *Roseburia* (all probiotic type bacteria) ↓ *Bacteroides* ↓*Carnobacteriaceae* ↑*Moyella*, *Peptococeaceae*, and *Ruminococcaecea* ↓ *Anaerotruncus, Dehalobecteriaceae*, *Blautia* and *Coprococus* ↑ *Streptophyta* order ↓ 4COD-2 ↓ *Bacteroidetes, Proteobacteria*, and *Tenericutes* ↓ *Alphaproteobacteria* and *Gammaproteobacteria*	^63^
**Black raspberries (*Rubus occidentalis*) (BRB)**	**32, 4 to 5-week-old, male Fischer 344 rats** **n = 8, control** **n = 8, 5% whole BRB powder** **n = 8, 0.2% BRB anthocyanins** **n = 8, 2.25% BRB derived residue** **Fecal material collected at weeks 0, 3 and 6.**	6 weeks	Bacterial DNA in feces was extracted using powerbead tubes V1–V3 regions of the bacterial 16 S gene were amplified Roche 454 pyrosequencing Data processing using QIIME	time-dependent changes in bacterial diversity within each diet whole BRB powder: ↑ *Anaerostipes*, *Ruminococcus, Akkermansia* and *Coprobacillus*, ↓ *Acetivibrio* at weeks 3 and 6 transiently ↑ *Allobaculum*, and a transiently ↓ *Anaerotruncus* at week 3 BRB-derived anthocyanin fraction: ↑*Anaerovorax* and *Dorea*, ↓*Bifidobacterium* and *Lactococcus* at weeks 3 and 6 transiently ↑*Asaccharobacter*, and a transiently ↓*Prabacteroides* at week 3 BRB-derived residue fraction: ↑ *Anaerotruncus, Coprobacillus, Desulfovibrio, Victivallis*, and *Mucispirilum* ↓ *Streptococcus, Turicibacter*, and *Acetivibrio* at weeks 3 and 6 transiently ↑ *Ethanoligenens* and transiently ↓ *Bifidobacterium* at week 3	^64^
**Broccoli**	Fischer 344 rats, weighing 120–140 g Study 1, 18 rats, 10% Cooked Broccoli (CB) Diet: n = 3, 0 d n = 3, 4 d n = 3, 1 ds n = 3, 7 d n = 3, 2 d n = 3, 14 d Study 2, 32 rats: n = 8, control n = 8, cooked broccoli (CB) n = 8, glucoraphanin (GRP) n = 8, CB-H diet (no GRP) Study 3, 9 rats: n = 3, control n = 3, raw broccoli 4 d n = 3, raw broccoli 4 d+ control 3 d Cecal contents collected.	14 d (with sampling at d0, 1, 2, 4, 7, and 14)	Total DNA was extracted from cecal contents using QIAmp DNA stool Mini Kit PCR 16 S rRNA gene sequencing (Illumina MiSeq) QIIME, GreenGenes database	↑OTUs, Chao1, Shannon and Simpson indices in rats fed cooked broccoli for ≥4 d. Six genera, mostly from the order *Clostridiales* (↓*Blautia*, ↓*Clostridium*, ↓*Dorea*, ↑*Ruminococcaceae* (family, genus not assigned) and ↑*Oscillospira*) significantly changed in abundance after CB feeding ≥4 d Non-GRP components of broccoli are responsible for the new cecal microbial community structure.	^65^
**Polished rice (PR), refined wheat (RW), unpolished rice (UPR) and whole wheat (WW)**	50, 7-week-old, male Sprague–Dawley rats n = 10, basal diet-fed group n = 10, PR group (50% PR+50% basal diet) n = 10, UPR group (50% UPR+50% basal diet) n = 10, RW group (50% RW+50% basal diet) n = 10, WW group (50% WW+50% basal diet) Ileal, cecal and colonic content collected, frozen in liquid nitrogen and stored at −80°C.	6 weeks	DNA Extraction using the CTAB/SDS method 16 S rRNA gene sequencing (Illumina MiSeq) QIIME	UPR and WW: ↓*Firmicutes*/*Bacteroidetes* *Lactobacillus* in rats under wheat diets (38% in the RW group and 41% in the WW group) was significantly higher than that under rice diets ↑*Akkermansia* in RW group compared to PR group	^66^
**Whole rye (WR)**	24 male Wistar rats (75–100 g) n = 12, 50% whole rye (WR) n = 12, 50% refined rye (RR) Feces sampled at the start and end of the experiment. The cecal content sampled at the time of sacrifice and frozen at −80°C.	12 weeks	DNA extraction using Qiagen’s DNA Stool Kit DNA quantification using a NanoDrop ND-1000 spectrophotometer Microbial community analysis performed using the Human Gut Chip (HuGChip)	Whole rye: feces: ↑ diversity ↓*Firmicutes/Bacteroidetes* ratio ↓ proportions of uncultured *Clostridiales* cluster IA and *Clostridium* cluster IV	^67^
**Polyphenolic compounds**					
**Green tea polyphenols (GTP)**	72, 6-month-old, ovariectomized female Sprague-Dawley rats Sacrificed after 3 months: n = 12, Control group n = 12, 0.5% GTP n = 12, 1.5% GTP Sacrificed after 6 months: n = 12, Control group n = 12, 0.5% GTP n = 12, 1.5% GTP Colon contents collected and stored in −80°C.	6 months	DNA extraction using Qiagen Fast DNA Stool Mini Kit PCR sequencing on Ilumina MiSeq Metagenomic analysis SMC-seq, MEGAHIT QIIME	3 months: ↓ 2 *Bacteroidetes* OTU (genus CF231 and *Bacteroides*) ↑ 6 *Firmicutes* OTU (6 of *Clostridiales* order; family *Ruminococcaceaae* and *Lachnospiraceae*) 6 months: ↓ 7 *Firmicutes* OTU (5 of *Clostridiales* order (*↓Peptostreptococcaceae family) and* 2 of *Erysipelotrichales* order) ↓ 1 *Bacteroidetes* OTU (S24-7) ↑ 3 *Bacteroidetes* OTU (1 of *Bacteroidaceae*, 2 of S24-7) ↑ 1 *Proteobacteria* OTU of *Desulfovibrionales* ↑ 1 *Firmicutes* OTU (genus *Oscillospira*)	^68^
**Grape seed proanthocyanidins (GSPE)**	27 female Wistar rats weighing 180–200 g n = 9, control group n = 9, gallic acid n = 9, GSPE Cecal content collected, frozen in liquid nitrogen and then stored at −80°C.	8 d	gDNA from cecal content of rats was extracted using a Fast DNA Stool Mini Kit (QIAGEN) PCR sequencing on Illumina MiSeq QIIME, GreenGenes database	Phylum: ↓ *Firmicutes* ↑ *Bacteroidetes* and *Proteobacteria* Families ↑S24-7, ↑*Bacteroidaceae* and ↑*Porphyromonadaceae* (class *Bacteroidia*) ↑ *Alcaligenaceae* (*Betaproteobacteria*) ↑ *Veillonellaceae* (*Clostridia*) ↓ *Ruminococcacea* ↓ *Dehalobacteriaceae* Genera: ↑*Bacteroides*, ↑*Parabacteroides*, ↑*Sutterella* ↑*Phascolarctobacterium* ↑*Bilophila* (vs control) ↓*Ruminococcus*	^69^
**Phenolic compounds (PC) rich grape pomace extracts**	30, 2-month-old, male Wistar rats n = 6, 2.5 mg/kg/d PC n = 6, 5 mg/kg/d PC n = 6, 10 mg/kg/d PC n = 6, 20 mg/kg/d PC n = 6, control Fecal samples collected at 6 and 14 months posttreatment and stored at −80°C.	14 months	DNA was extracted using ISOLATE Fecal DNA Kit Quantitative analysis of intestinal microbiota of the different bacterial genera was carried out by qPCR	*↑Bifidobacterium* (PC 2.5 and PC 5) After 14 months of treatment all concentrations of PC abolished the increase of *Clostridium sensu stricto* (cluster I).	^70^
**Hesperidin (HD) and its aglycone Hesperetin (HT)** **major flavonoids in citrus fruits**	21, four-week-old male Wistar rats n = 7, control diet n = 7, 0.5% HT diet n = 7, 1.0% HD diet Feces collected and stored at −40°C.	3 weeks	DNA extraction Quantitative real-time PCR of 16 S rRNA genes Fecal samples of rats were analyzed by targeting the bacterial 16 S rRNA genes using a terminal restriction fragment length polymorphism (T-RFLP) technique	↓proportion of *Clostridium* subcluster XIVa ↑Clostridium clusters IV and XVIII	^71^
**Persimmon (*Diospyros kaki Niuxin*) tannin (PT)**	48 male Sprague-Dawley rats, weighing 120–140 g Normal diet: n = 6, control n = 6, low PT (LPT)+ 50 mg/kg·BW PT n = 6, medium PT (MPT) +100 mg/kg·BW PT n = 6, high PT (HPT) + 200 mg/kg·BW PT +4 groups high fat diet Cecum content collected and frozen at −80°C.	4 weeks	DNA extraction using QIAamp® DNA Stool Mini Kit Real – time PCR, Rotor-gene 6 software Sequencing (MiSeq system, Illumina)	LPT, MPT: *↓Firmicutes* ↑*Bacteroidetes* *LPT:* *↓Firmicutes*/*Bacteroidetes* ratio *↑Prevotella, ↓Bacteroides, ↓Phascolarctobacterium, ↓E. coli* ↑total *Lactobacillus* count *HPT:* *↑Proteobacteria* *↓Bacteroidetes/Proteobacteria* ratio	^72^
**Probiotics and prebiotics**					
**Probiotics *L. acidophilus* NCFM and *B.lactis* Bi-07 in combination with polysaccharides (*Lyceum barbarum* polysaccharides, *Poria cocos* polysaccharides and *Lentinula edodes* polysaccharides)**	24, 21-d-old, male Sprague-Dawley rats n = 12, control group n = 12, probiotics with polysaccharides Feces samples collected and stored at −80°C.	28 d	Feces total DNA was extracted with a MOBIO PowerSoil®DNA Isolation Kit 16 S rRNA gene amplification (PCR) sequencing (Hiseq 2500 platform) QIIME, GreenGenes database	significantly changed: Phylum level: ↑*Actinobacteria* Order level: ↑*Bifidobacterials, Lactobacillales, Erysipelotrichales* and *Aeromonadales* ↓*Clostridiales* Family level: ↑*Bifidobacteriaceae*, S24_7, *Lactobacillaceae, Aerococcaceae, Staphylococcaceae, Erysipelotrichaceae* and *Aeromonadaceae* ↓*Enterococcaceae, Rikenellaceae* and *Porphyromonadceae* genus level: ↑*Bifidobacterium, Lactobacillus, Allobaculum, Alcaligenes* and *Oligella* ↓*Anaerostipes, Enterococcus* and *Parabacteroides* species level: ↑*Bifidobacterium pseudolongum, Lactobacillus salivarius, and Lactobacillus reuteri* ↓ *Alcaligenes faecalis, Bacteroides ovatus* and *Bacteroides eggerthii*	^73^
**Blueberries fermented with the tannase producing bacteria L. plantarum DSM 15313**	54, 12–13-week–old, male Sprague Dawley rats 3 groups-hypertensive state ± Healthy rats: Fed two types (A and B) of freeze-dried blueberry powder fermented over night after incubation with L. plantarum DSM 15313 with additional10^9^ cfu/day of L. plantarum DSM 15313. Product A was fermented to a higher extent than product B. n = 9, control n = 9, product A+ bacteria n = 9, product B+ bacteria Cecal content collected.	4 weeks	Terminal restriction fragment length polymorphism (T-RFLP) SYBR green qPCR	Product A and B ↓ TRF303, which was putatively identified as bacteria belonging to *Lachnospiraceae* in another study ↓T-RF 228 ↑T-RF 88, 91, 92 which were identified as *Parabacteroides*-like and *Bacteroides*-like T-RF 132 was only detected in product-receiving groups product B: ↓*C. leptum* group ↓ *Desulfovibrio*	^74^
**Galactooligosaccharide (GOS)–** **fish peptide (FP) conjugates**	40, 3-week-old male Sprague-Dawley rats n = 8, CK – control n = 8, GOS n = 8, FP n = 8, GOS/FP mixture n = 8, glycoconjugates (G-GOS/FP) Feces collected.	21 d	DNA extraction (TIANamp Stool DNA Kit) PCR Illumina 16 S rRNA sequencing QIIME	G-GOS/FP group compared to the CK group: ↓ alpha-diversity ↑ *Actinobacteria* phylum ↑ *Coriobacteriaceae* and *Veillonellaceae* family G-GOS/FP group compared to the GOS/FP group: ↑ *Anaerovibrio* ↑*Prevotella-9, Collinsella* (most differential bacteria) ↓ *Alloprevotella, Holdemanella*	^75^
**Fructans** **from *Agave salmiana***	15, 4-week-old male Wistar rats n = 5, commercial diet n = 5, commercial diet added with dried extract of *A. salmiana* n = 5, commercial diet added with symbiotic formulation (dried extract of *A. salmiana*+powder of encapsulated *Bifidobacterium animalis subsp. lactis*) Feces collected.	12 weeks	bacteria count (Microbial growth was determined as CFU g^−1^ of lactic acid bacteria (LAB) in feces after 72 h incubation)	↑ *Lactobacillus spp*. and *Bifidobacterium spp*. (CFU g^−1^) in both test groups (approximately 4-log increase was observed in the group with symbiotic food)	^76^
**Feruloylated oligosaccharides** **(FOs) from maize bran**	45, male Sprague–Dawley rats, weighing 160–170 g n = 9, normal feed n = 9, + 300 mg/kg BW/d xylooligosaccharides (XOS) n = 9,+ 300 mg/kg BW/d XOS + 12 mg/kg BW/d ferulic acid (FA) n = 9, + 300 mg/kg BW/d FOs n = 9, + 600 mg/kg BW/d FOs Feces collected and stored at −80°C.	5 weeks	DNA extraction using TIAN amp Stool DNA kit PCR amplification sequencing on the Illumina GAIIx platform reference base from Ribosomal Database Project	Feruloylated oligosaccharides showed even higher prebiotic activity than XOS FOs: ↑bacterial richness and diversity ↓ ratio of *Firmicutes* to *Bacteroidetes* ↑ *Bacteroidetes*, *Proteobacteria, Actinobacteria, Lactobacillus* and *Ruminococcus* ↓*Clostridia*	^77^
**Pectins (four types)**	40, male Wistar rats (300 ± 10 g) n = 8, control group n = 8, 3% (w/w) low-methyl esterified citrus pectin (LMP) n = 8, 3% (w/w) high-methyl esterified citrus pectin (HMP) n = 8, 3% (w/w) sugar beet pectin (SBP) n = 8, 3% (w/w) soy pectin (SSPS) Cecal and colonic digesta collected separately.	7 weeks	Microbial DNA was extracted from digesta using a Repeated Bead Beating protocol plus column purification DNA was quantified using a NanoDrop 2000 spectrophotometer PCR 16 S rRNA gene amplification and Illumina MiSeq sequencing QIIME	pectin supplemented diets: ↑*Firmicutes* and ↓*Bacteroidetes* in both cecal and colonic digesta effects more pronounced in cecum than in colon *↑ Lactobacillus* (LMP, SBP) *↑ Lachnospiraceae* (SBP) *↑ Incertae_Sedis* in the family *Lachnospiraceae* and ↓unclassified genus in the family *Peptostreptococcaceae, ↓Akkermansia* relative abundance *(SBP, SSPS)* ↓Unclassified genus in the family *S24-7* (HMP, SBP)	^78^
**Fermentable carbohydrates**	24 male BioBreeding rats (28 to 42-d-old) n = 8, cellulose (C: 5% w/w) n = 8, wheat bran (WB: 5% w/w) n = 8, high amylose maize starch (RS: 5% w/w resistant starch) Fecal pellets collected at the end of the feeding trial (d 28). Cecal contents collected during necropsy following completion of the balance phase (d 42).	6 weeks	Community DNA isolated using Qiagen DNA Stool Isolation Kit Bacterial tag-encoded FLX amplicon sequencing Silva database Phylotypes classified using the Ribosomal Data-base DNA Shotgun metagenomic library construction and sequencing (Roche GS-FLX Titanium sequencing) MEGAN	Cecal communities were dominated by the phylum *Firmicutes* with higher abundance of *Lachnospiraceae* compared to feces. In feces the community structure was shifted toward the phylum *Bacteroidetes.*	^79^
**Dietary fibers (inulin, resistant starch and citrus pectin)**	18 5/4-month-old male wild-type Groningen rats n = 18, acclimatization week 14 d: n = 6 inulin (10%) n = 6 resistant starch (10%) n = 6 citrus pectin (3%). n = 18, 1-week wash-out period Fresh fecal samples collected from the bedding and stored at −20°C.	14 d with sampling points: T0 (end of acclimatization period), T1 (after 1 week of supplementation) T2 (after 2 weeks of supplementation) T3 (after a wash-out period)	DNA extraction using the QIAamp DNA Stool Mini Kit (Qiagen) Partial 16 S rRNA gene sequences amplified using primer pair Probio_Uni and/Probio_Rev, targeting the V3 region of the 16 S rRNA Illumina MiSeq sequencer QIIME	↑*Bacteroidetes* ↓Firmicutes Inulin: Within Bacteroidetes phylum: ↑ *Prevotellaceae* family ↑ *Parabacteroides spp*. ↓ *Bacteroidales* ↑ *Proteobacteria* phylum Within *Firmicutes* phylum: ↓*Lachnospiraceae* *↓ Clostridiales* *↓ Ruminococcaceae* *↑ Allobaculum spp*. Resistant starch: *↑Prevotellaceae* *↓Lachnospiraceae* *↓ Clostridiales* *↑ Ruminococcus* 2 group *↓Ruminococcus 1* *↓Ruminococcaceae UCG_002* Citrus pectin: ↑*Prevotellaceae* NK3B31 group ↑*Ruminococcacea*e UCG-005 *↓ Stomatobaculum spp. (Lachnospiraceae family)*	^37^
**Minerals**					
**Marine mineral blend (seaweed and seawater-derived, rich in bioactive calcium, magnesium and 70 other trace elements) (MMB)**	30 7-8-week old male Sprague-Dawley rats n = 10, control group, standard chow n = 10, 0.1% MMB-supplemented chow n = 10, 0.2% MMB-supplemented chow Cecal content collected and stored at −80°C.	6 weeks	DNA extraction using QIAmp Fast DNA Stool Mini Kit PCR Ilumina MiSeq sequencing platform QIIME	↑ diversity ↓ phylum TM7 (associated with IBD, Chron’s disease) in 0.1% MMB ↑*Proteobacteria* (linked to inflammation and metabolic syndrome) in supplemented groups ↑ phylum RF3 in 0.2% MMB ↑ *Ruminococcaceae, Clostridaceae* (SCFA production) ↑ *Christensenellaceae* (lean BMI) ↑ *Porphyromonadaceae* (protective effect on gut health)	^80^
**Methylxanthines**					
**Cocoa theobromine**	21 3-week-old Lewis rats n = 7, reference group (RF) n = 7, standard diet with 10% of natural Forastero cocoa containing 0.25% theobromine (CC) n = 7, standard diet including 0.25% of theobromine (TB) Feces collected at d 0, 8, and 15, frozen at −20°C.	15 d	Quantification of fecal microbiota by fluorescence in situ hybridization (FISH) coupled to flow cytometry (FCM) IgA-coated bacteria quantification metagenomics analysis: FastDNASPIN Kit 16 S rDNA sequencing in Ion Torrent platforms QIIME Greengenes reference database	Theobromine group: FISH-FCM results: ↓ *E. coli* ↓ *Bifidobacterium spp., Streptococcus spp., Clostridium histolyticum-C. perfringens* ↓ *Clostridium, Bacteroidaceae-Prevotellaceae* ↓ *Firmicutes* metagenomics analysis: ↑ proportion of *Tenericutes* phylum ↑ proportion of the *Erysipelotrichaceae* family (*Firmicutes* phylum), *Ralstonia sp*. (*Proteobacteria* phylum) and one bacterium of the *Mollicutes* class (*Tenericutes* phylum) *Candidatus Arthromitus* (*Firmicutes* phylum,*Clostridia* class, able to induce adaptive immune responses in the gut) found exclusively in the TB group while *Ruminicoccus flavefaciens* (*Firmicutes* phylum) disappeared ↓ IgA-coated bacteria	^81^
**Fungi**					
**Reishi mushroom (*Ganoderma lingzhi*) water extract**	24 male 3-week old Sprague-Dawley rats n = 8, control diet n = 8, 5% water extract from the reishi mushroom (*Ganoderma lingzhi*) (WGL) n = 8, 5% water extract from auto-digested reishi *G. lingzhi* (AWGL) Cecum content collected and stored at-80°C.	3 weeks	DNA was isolated from the cecal digesta using the UltraClean™ Fecal DNA extraction kit Bacterial groups were quantified by qPCR	WGL and AWGL treatments ↓*Clostridium coccoides* and *Clostridium leptum* per g of digesta ↑ *Akkermansia muciniphila* per g of digesta and per total digesta ↑ *Bacteroidetes* per total digesta ↑ *Enterobacteriaceae* per g of digesta and per total cecal digesta	^82^
**Polysaccharide from mycelia of *Ganoderma lucidum***	20 male Sprague-Dawley rats, weighing 198 ± 15.7 g n = 10, control group (CON) n = 10, *Ganoderma lucidum* polysaccharide group (GLP) Cecal contents collected and stored at −80°C.	21 d	Bacterial total genomic DNA extracted using the E.Z.N.A. Genomic DNA Isolation Kit PCR Roche Genome Sequencer GS FLX Titanium platform QIIME	↓*Firmicutes/Bacteroidetes* ratio ↓*Proteobacteria* 37 OTUs were significantly different (↑S24-7, ↑SMB53, ↑*Rikenellaceae*, ↓*Allobaculum*, ↓Rc4-4 and ↓*Ruminococcaceae*)	^83^
**Vegetable oils**					
**Camellia Oil (*Camellia oleifera Abel*., CO), olive oil (OO) and soybean oil (SO)**	30 male 6-week-old Sprague-Dawley rats n = 18, SO group n = 6, CO group n = 6, OO group Fecal samples collected and stored at −80°C.	20 d	DNA Extraction (Qiagen DNA Mini Kit) 16 S rRNA Sequencing (llumina MiSeq 2000 sequencer with a MiSeq Reagent Kit v 3)	Compared to SO and OO, the intake of CO increased the ratio of *Firmicutes/Bacteroidetes*, the α-diversity, relative abundance of the *Bifidobacterium*, and reduced *Prevotella*	^84^
**Plants**					
**Aloin, a component of the *Aloe vera* plant leaf**	Male F344/N Nctr rats n = 10, control group n = 10, 6.95 mg/kg n = 10, 13.9 mg/kg n = 10, 27.8 mg/kg n = 10, 55.7 mg/kg n = 10, 111 mg/kg n = 10, 223 mg/kg n = 10, 446 mg/kg of drinking water Feces collected.	13 weeks	DNA extraction (PowerSoil® DNA Isolation kit) next generation sequencing of the PCR amplified V3/V4 region of the 16 S rRNA gene (Illumina MiSeq) QIIME	↑*Bacteroidetes* phylum in doses of 111, 223, and 446 mg/kg (mostly due to family *Prevotellaceae* and S24-7) ↑*Verrucomicrobia* phylum in doses of 223 and 446 mg/kg ↓*Firmicutes* phylum in doses of 111, 223, and 446 mg/kg(mostly due to family *Ruminococcaceae*, *Lachnispiraceae*) ↓class *Clostridia* in the 111, 223, and 446 mg/kg	^85^
**Natural pigments**					
**Natural pigment violacein from *Chromobacterium violaceum***	16, 2-month-old, male Wistar albino rats n = 6, 50 μg/mL violacein n = 6, 500 μg/mL violacein n = 4, control group Whole intestinal content collected.	One month	DNA extraction using QIAamp DNA Stool Minikit and MoBio PowerSoil DNA Isolation kit PCR amplificaton Denaturing Gradient Gel Electrophoresis Pyrosequencing QIIME PICRUSt	Higher richness ↑ *Firmicutes* ↓ *Proteobacteria* ↑ *Lactobacillaceae* family *Bacilli, Clostridia, Bacteroidia* among dominant classes in low violacein group *Actinobacteria, Coriobacteria* in high violacein group	^86^

↑- increased abundance, ↓ -decreased abundance, BW-body weight

The experimental design in research of microbiota initiates often with animals undergoing an acclimatization period, usually for 1 week. During this period rats retain their habits and are kept on a normal chow diet. These rats represent a negative control of themselves, acting as the baseline for further microbiota evaluation.^37^

A typical microbiota experiment is designed in such a way that microbiota diversity and species richness between different experimental groups of rats are compared. One group of animals has not been treated with a nutritive supplement under evaluation, this is the control group, and one or more groups of animals are exposed to the assessed nutrient (treated groups), often at different doses. The diet of treated rats has the same composition as the diet of control rats but supplemented with the assessed nutrient. In majority of the studies presented in Table 4 the assessed nutrient was mixed with the feed. In fewer occasions, the supplement was added to drinking water as in (1) the study conducted by Wang *et al*. where treatment groups were given daily freshly prepared distilled water mixed with green tea polyphenols, (2) in the study by Chacar *et al*. where treatment groups were given different concentrations of phenolic compounds in the drinking water and (3) in Boudreau *et al*. study where aloin was administered to rats at different doses in drinking water.^68,70,85^ In the study conducted by Jin *et al*. the rats in treatment group received the polysaccharide *Ganoderma lucidum* in aqueous suspension daily by oral administration.^83^ Also, the assessed nutrient can be given to treatment groups by gavage as it was the case in the study of Casanova-Martí *et al*. where treatment groups were given grape seed proanthocyanidins or gallic acid 1 h prior to chow replacement by gavage, using tap water as vehicle.^69^ Gastric administration of the assessed supplement was also performed in the study of Ou *et al*. where the effects of feruloylated oligosaccharides from maize bran on the microbial diversity and profiles were investigated in rat feces and in the study of Lee *et al*. where camelia oil, olive oil, and soybean oil were administered to rats by gastric gavage.^77,84^ Also, in the study of Pauer *et al*., rats received violacein directly in the mouth, twice a day, by gavage for a month.^86^

Typically, six to 12 animals are assigned to each group although variations have been seen. Therefore, the smallest number of animals in a group was three, while the largest groups comprised 18 animals. Moore and Stanley highlighted factors that need to be taken into consideration when designing an animal trial aimed to investigate the gastrointestinal tract microbiota in the context of inflammation studies. They noted that unlike in traditional studies of immune mechanisms and inflammatory diseases in mouse models where the group sizes usually include 3 to 12 animals, larger treatment groups are necessary in microbiota experiments to achieve sufficient statistical power to draw valid conclusions.^93^ This is mainly due to the inherent variability in microbiota between animals as well as to the temporal variation and strong responsiveness to diverse environmental stimuli.

The effect of nutritional supplementation on the composition of gut microbiota in an experiment is evaluated after a certain period of time. Most treatments presented in the literature continued for 3, 4, or 6 weeks; however, there were several studies focused on short-term and long-term nutritive modulation.^37,65,68-70^ For example, Casanova-Martí and coworkers evaluated the effect of grape seed proanthocyanidins on the rat microbiota during an 8-d trail.^69^ In addition, in a study by Ferrario *et al*. where the effects of three different dietary fibers on rat fecal microbiota were evaluated, the grouping of the samples after a period of intervention of 1 week was indicative for different effects induced by dietary ingredients.^37^ The study conducted by Liu and coworkers evaluated the effect of dietary broccoli with a multiple-sampling points (after 1, 2, 4, 7, and 14 d of treatment). The latter study demonstrated that changes in microbiota were present already after 4 d of treatment.^65,69^ In contrast, Wang *et al*. studied the effects of green-tea polyphenols over a period of 6 months while the study of Chacar *et al*. who investigated a long-term intake of phenolic compounds had an even longer study period of 14 months.^68,70^ The study of Ferrario *et al*. had a wash-out period where all animals returned to the standard chow diet without added substances, for 1 week after the end of the experiment in which dietary fibers were supplemented and it showed a reversion back to baseline microbiota.^37^ Longitudinal studies that incorporate samples from the same habitat over time might provide more accurate conclusions rather than simple cross-sectional studies that compare ‘snapshots’ of two sample sets.^94^

At the end of the defined experimental period, sampling is conducted and fecal or intestinal material is collected followed by microbiota analyses. Fecal material, freshly voided or collected from bedding is sampled in sterile tubes while intestinal content is collected at the time of sacrifice, dissected, and also sampled in sterile containers. In the study of Ferrario *et al*. fresh fecal samples were collected manually from a clean sawdust bedding for each animal, at most 1 h after deposition.^37^ In the study of Pauer *et al*. whole intestinal content was collected, while Han *et al*. sampled ileal, cecal, and colonic content separately.^66,86^ The samples are then stored at −80°C (in some cases −40°C and −20°C) until further analysis.

### Insight into rat intestinal microbiota modulation by food supplements

**Figure 1.**
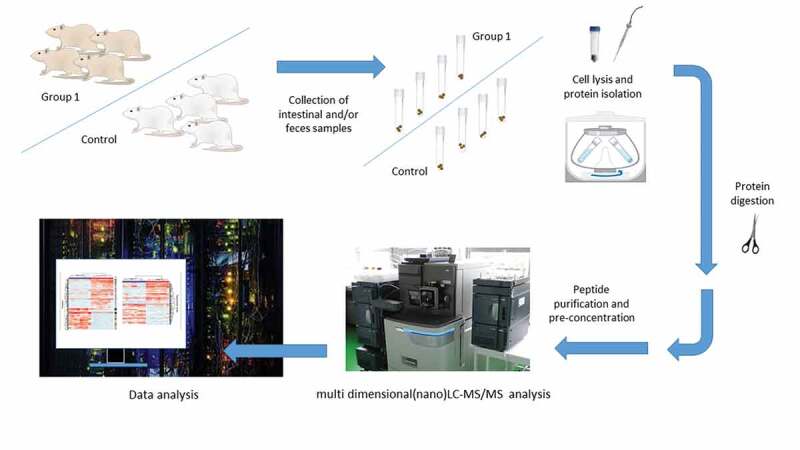
An example of the metaprotoemics workflow based on LC-MS/MS methods.

The usage of food supplements has increased in recent years even though scientific data on their effects *in vivo*, including effects on the microbiota, have been often vague.^95^ This is why studies on animals may provide a good rationale for further, translational studies in human subjects. Rat models have been also used in studies that rely on genomics, proteomics, and metabolomics methods for the identification of microbiota members, mainly bacterial population, and the elucidation of functional changes. Main genomics methods used for this purpose are polymerase chain reaction (PCR) used in conjunction with high-throughput 16 S ribosomal RNA (rRNA) sequence analysis, that is, the second generation of sequencing technologies (Table 4).^95^ Recently, the third generation of sequencing approaches is also applied, however, less frequently (Table 4), while real-time quantitative PCR (RT-qPCR) is rarely employed in the (targeted) estimation of bacterial loads, particularly for selected bacterial species.^95^ These approaches are known as metagenomics methods preformed according to the widely agreed standards.^63^ Genomics methods for the analysis of microbiota have reached a satisfactory level of technological maturity,^96–98^ as well as metaproteomics and metabolomics approaches for a comprehensive assessment of taxonomic composition and, particularly, its corresponding functionality. However, the usual metaproteomics experimental workflow includes multiple steps that require additional standardization (Figure 1). These steps include isolation of bacteria from feces or intestinal content, bacterial protein isolation, tryptic digestion, and the identification/quantification of proteins by liquid chromatography coupled with tandem mass-spectrometry (LC-MS/MS).^99^ Latter approach aimed to characterize the metaproteome is based on the comparison of experimentally obtained protein data with genomics information. This often implies an experimental design comprising both metagenomics and metaproteomics analyses from the same samples.^33,100^ Such – omics-based methods are crucial for the microbiota identification and have been exploited to analyze the effects of food, nutrients, and food supplements on the microbiota status of the rats.

The key effects on the rat gut microbiota after consummation of different nutritional supplements are summarized from the literature in Table 4. The results presented in Table 4 are denoting changes that were statistically significant or otherwise emphasized by the authors as noteworthy.

Different foods, for example, fruits, vegetables, nuts, pulses, and cereal grains exerted their beneficial effect in part due to their polyphenolic or dietary fiber content. The consumption of walnuts, as the consumption of broccoli, increased the overall species diversity in the gut microbial communities.^63,65^ For instance, walnut diet increased the abundance of *Firmicutes* and in parallel decreased the abundance of *Bacteriodetes*, making the *Firmicutes* predominant microbe phyla in the descending colon. Moreover, probiotic-type bacteria including *Lactobacillus, Ruminococcaceae*, and *Roseburia* were enriched, while some others, like *Bacteroides* and *Anaerotruncus* were significantly reduced.^63^ Interestingly, the latter changes in microbial composition may not be followed by significant changes in rat body weight and food intake, between the control and treated groups. Nevertheless, as the authors emphasize the addition of walnuts to the diet may shift the relative abundance of the functional capacities of the microbial communities. In total, 12 KEGG (Kyoto Encyclopedia of Genes and Genomes) metabolic pathways may be affected; the most of them involved in the amino acid and omega −3 and −6 fatty acid metabolism.^63^

Several supplements, for example, **lentil, unpolished rice, and whole wheat** reduced the *Firmicutes/Bacteroidetes* ratio.^62,66^ In human and in animal studies, higher *Firmicutes*/*Bacteroidetes* ratio is linked to higher body mass index (BMI).^101,102^ In line, body weight gain reported for the rats fed with unpolished rice and whole wheat was lower compared to other groups fed by polished rice and refined wheat. For the same beneficial diets, an increased content of total short-chain fatty acids (SCFA), like acetate and butyrate in cecal and colonic digesta were reported.^66^ The latter observation is normal for bacteria that ferment fibers and is required for optimal health, frequently attributed to the wide-ranging impacts of SCFA on the host physiology.^103–106^ In addition, Han *et al*. found beneficial bacteria like *Lactobacillus* and *Akkermansia* significantly increased in microbiota community of wheat-fed rats compared to in rice-fed rats.^66^ The both species of the latter human intestinal bacteria are known to facilitate fermentation of indigestible carbohydrates, originating from dietary fibers, resistant starches, and non-starch polysaccharides.

Ounnas *et al*. reported microbiota changes in feces after consumption of whole rye, while no changes were evident in cecum microbiota.^67^ Nevertheless, as author indicate this was one of the first studies in which the consumption of whole rye and its beneficial health effects were investigated, and results showed major biological modifications. Precisely, next to gut modifications like decreased *Firmicutes/Bacteroidetes* ratio, rats with whole rye diet had significantly increased n-3 long-chain fatty acids (LCFA) in their plasma and liver. The specific diet particularly influenced the metabolism of eicosapentanoic and docosahexanoic acids, while the content of SCFA was decreased, both in cecum and feces.^67^

**Polyphenolic compounds**, such as tannins and hesperidin or hesperetin and polyphenolic mixtures, like green tea polyphenols (GTP), grape seed proanthocyanidins extract (GSPE), grape pomace extract have been studied for their effect on the rat gut microbiota. For example, the *Firmicutes/Bacteroidetes* ratio decreased after consumption of GSPE and persimmon tannin, while the abundance of *Bacteroidetes* phylum increased after supplementation with green tea polyphenols.^68,69,72^ Indeed, the long-term treatment (6 months) with GTP significantly decreased the biodiversity in a dose-dependent manner at Sprague-Dawley rats. Moreover, similar patterns were observed at both sampling times, at the end of month 3 and 6.^68^ Along with increased *Bacteroidetes* phylum, Wang *et al*. reported enrichment for *Oscillospira*. The effect of GTP intake may be further evaluated on obese gut microbiome as discussed changes in the gut microbiome were previously associated with leanness in humans and animals.^107^ Another beneficial effect was the decrease of *Peptostreptococcaceae* which was linked to colorectal cancer phenotype in a study of Ahn *et al*.^108^

Interestingly, similar results were obtained after only 8-d (short-term) treatment with GSPE, however, the latter study in a systematic approach evaluated also the effect of polyphenolic compounds on enteroendocrine secretions in female rats. Consequently, when polyphenolic mixtures are used as supplements it is difficult to differentiate which compounds are immediately absorbed, and which remain in the lumen, potentially causing gut modulation and, subsequently, indirectly changing the host’s health status. Due to present limitations of applied analytical approaches and techniques and the complexity of human/animal organisms, it is still not possible to observe the impact separately. In their study of proanthocyanidins, Casanova-Martí *et al*. defined several new target taxonomic groups that are modulated by proanthocyanidins intake, these are *Sutterella, Pharscolarctobacterium, Parabacteroides, Bilophila*, and *Ruminococcus*.^69^ The increase in S24-7 family in the latter study is in accordance with the results of the previous study involving apple procyanidins in mice.^109^ Next to microbial shifts, Casanova-Martí *et al*. hypothesized that observed gut modulation may correlate with metabolic and morphometric variables. Indeed, their study confirmed correlation between the gut modulation and systems effect, specifically, the reduction in cecal butyrate amount as well as the increased level of plasma glucagon-like-peptide-1.^69^ In other word, the authors suggest that specific changes in microbiota caused by GSPE treatment may be linked to the modulation of plasma triacylglycerol, adiposity, and enterohormone secretion. Noteworthy posttreatment effects on the gut rat microbiota composition after long‐term (14 months) intake of grape pomace extracts rich with phenolic compounds were reported. Precisely, quantitative analysis of intestinal microbiota by qPCR revealed selective modulation, for example the growth inhibition of *Clostridium (*cluster I) 14 months posttreatment and the enhanced growth of probiotic *Bifidobacterium* 6 and 14 months posttreatment, compared to control and young groups.^70^ The authors of the latter study emphasized that the second presented microbiota modulation was dose-specific, that is, the concentrations of phenolic compounds above 5 mg/kg/d did not result in such beneficial modulations. In general, the abundance reduction of *Bifidobacterium* was reported with age-related changes in the gut microbiota; therefore, phenolic compounds might have a protective effect on gut bacterial population, and even modulate outcomes of aging. Decrease in *Clostridium* was noticed also after supplementation with hesperidin and hesperetin, major flavonoids in citrus fruits, that significantly decreased the ratio of *Clostridium* subcluster XIVa.^71^ The long-term intake of polyphenolic components potentially inhibits age-related increase of *Clostridium*, but only after 14 months posttreatment, and the effect seems to be independent of the administrated dose.^70^ Finally, the study of Zhu *et al*., besides changes in the *Firmicutes*/*Bacteroidetes* ratio, showed that persimmon tannin when ingested at low doses may modulate the microbiota by increasing *Bifidobacterium sp*. and *Lactobacillus sp*., while decreasing *E. coli* and *Enterococcus*.^72^ As persimmon tannin is highly polymerized and, therefore, non-absorbable in the intestine, its effect after ingestion is local. However, previous studies on animal models showed anti-hyperlipidemic and cholesterol-lowering effects, which in the latter study were somewhat attributed to the changes in bacterial structure and SCFA metabolism.

When comparing the results of **probiotic and prebiotic** supplementation studies, it can be observed that microbial shifts share common patterns. For instance, the increase of the *Actinobacteria* phylum was evident in at least three studies, these were the studies in which rats were fed (1) with *Lactobacillus acidophilus* NCFM and *Bifidobacterium lactis B*i-07 in combination with polysaccharides,^73^ (2) food supplemented with galactooligosaccharide-fish peptide conjugates^75^ and (3) feruloylated oligosaccharides from maize bran.^77^ As one can expected, the latter type of feed supplementation may in general significantly modulate bacterial richness and diversity, and particularly increase the amount of probiotic bacteria. Specifically, Wang *et al*. reported an increase of *Bifidobacterium pseudolongum, Lactobacillus salivarius, and Lactobacillus reuteri* and in parallel a decrease of *Anaerostipes, Enterococcus*, and *Parabacteroides*.^73^ The authors unambiguously proved the importance of both probiotics and prebiotics in the maturation of healthy gut microbiota biological function. Moreover, the modulation of bacterial community resulted in elevated activities of digestive enzymes and several metabolism pathways (amino acid, energy, and SCFA-related), finally resulting in healthy progress of the weaning rats. On the other side, Ou *et al*. showed that feruloylated oligosaccharides from maize bran may exert a beneficial effect through multiple ways, that is by decreasing the ratio of *Firmicutes* to *Bacteroidetes*, increasing *Lactobacillus* and *Ruminococcus* and decreasing *Clostridia*.^77^ It was previously reported, that feruloylated oligosaccharides release ferulic acid after fermentation by gut microorganisms, which was recognized as doubled physiological function, as ferulic acid may exhibit antimicrobial activity versus different microorganisms.^110,111^ All recently enumerated modulations of the gut microbiota have been recognized as contributing to protection against diabetes.

After receiving fructans from *Agave salmiana* in the study of Jasso-Padilla *et al*., rats had an increase in *Lactobacillus spp. and Bifidobacterium spp*.^76^ Shifts of colonic microbiota composition induced by pectins were not as prominent as shifts of cecal microbiota in the study conducted by Tian *et al*. ^105^ In the cecal microbiota the rise in *Lactobacillus* was also present, specifically in groups receiving low-methyl esterified citrus pectin and sugar beet pectin.

Upon supplementation of marine **mineral blend**, rich in bioactive calcium, magnesium, and 70 other trace minerals, bacterial species diversity increased, specifically increased levels of *Proteobacteria* were also noticed.^80^ Therefore, seaweed and seawater-derived functional food may be considered as a reasonable supplement next to the high fat/high sugar “Western diet”. In another study, phylum TM7, associated with IBD and Chron’s disease, decreased in the group receiving lower concentration of the supplement, while *Ruminococcaceae* family associated with gut health increased for a group given a higher concentration of supplement. Families *Christensenellaceae*, associated with lean BMI, and *Porphyromonadaceae* were increased as well. In parallel, phylum *Proteobacteria* level increased and the latter observation was suggested as a potential diagnostic signature for dysbiosis and illness as it is known that *Proteobacteria* have a low abundance in the gut of healthy humans.^112^

After administration of theobromine, a **methylxantine from cocoa powder**, several changes that were exclusive for the rats fed with theobromine were noticed. For instance, *Candidatus Arthromitus* belonging to *Firmicutes* phylum, *Clostridia* class, known for inducing adaptive immune responses in the gut, was found only in the theobromine group, while *Ruminicoccus flavefaciens* disappeared.^81^ Besides, significantly lower counts of *Bifidobacterium* spp., *Streptococcus* spp., *Clostridium histolyticum C. perfringens* and *Escherichia coli* were seen in comparison with controls. All enumerated changes were reflected in enhanced generation of SCFA, mainly the butyric acid. Finally, the authors hypothesize that theobromine, both on its own and as part of a cocoa diet, may contribute to the lower proportion of IgA-coated bacteria.

Furthermore, *Ganoderma lingzhi* **mushroom**, used in traditional Chinese medicine, was evaluated for its effect on gut microbiota in rats.^109^ The treatment significantly reduced the numbers of *Clostridium coccoides* and *Clostridium leptum* per gram of digesta, while *Akkermancia muciniphila* and *Enterobacteriaceae* increased. In addition, the supplementation of polysaccharide from mycelia of *Ganoderma lucidum* decreased *Firmicutes/Bacteroidetes* ratio, *Proteobacteria* phylum and caused a significant change in 37 OTUs among which were S24-7, SMB53, *Rikenellaceae, Allobaculum*, Rc4-4, and *Ruminococcaceae*.^83^ Previously, increased diversity of *Clostridium coccoides* and *Clostridium leptum* was seen in microbiota profiles of patients with colon cancer and adenomatous polyposis.^113^ The both presented studies, emphasize the potential anti-colon cancer effect of mushroom extracts through firstly, modulating intestinal microflora, and secondly, a wide net of action, such as modulation of secondary bile acids, mucins, propionate, and regulation the intestinal barrier functions.

Rat gut microbiota changes after consummation of camellia olive and soybean **oil** were also reported.^84^ Moreover, the intake of camellia oil showed improved results in comparison to soybean and olive oil. Camellia oil modifies the composition of gut microbiota and alleviates acetic acid-induced colitis in rats. In more detail, the increased ratio of *Firmicutes/Bacteroidetes*, the species diversity, and the relative abundance of the *Bifidobacterium*, while reducing *Prevotella* was shown. Therefore, camellia oil is preferable treatment/preventive measure as it is able to reduce damage caused by antioxidant system induced by acetic acid, and finally may prevent the development of chronic inflammatory bowel disease. A component of Aloe vera **plant** leaf, aloin, may induce dose-related changes, for example, increased *Bacteroidetes* (mostly *Prevotellaceae* and S24-7) and *Verrucomicrobia* phylums and decreased *Firmicutes* (specially members *Ruminococcaceae* and *Lachnispiraceae)*.^85^ Moreover, the similarities in effects were observed for aloin and the Aloe vera whole leaf extract,^114^ including serious pathological changes leading to the increased incidences of adenomas and carcinomas of the rat cecum and large intestine. The latter findings suggest at caution when using Aloe vera latex laxative properties in humans and animals. Indeed, to achieve its purpose food/feed supplements must be further studied in order to hamper severe damage and increase resistance of pathogenic strains. For instance, violacein is a **natural** violet **pigment** produced by *Chromobacterium violaceum* with broad antibacterial, antiviral, antifungal, and antioxidant properties, however, its effect on rat gut microbiota of Wistar rats has been explored recently.^86^

## Conclusions

A link between gut dysbiosis and various diseases in humans has been observed, and the modulation of gut microbiota may be used for studies on prevention measure for various pathological states. The use of rats as disease models to study specific pathological states may be a valuable tool for determination of the relationship between intestinal dysbiosis and disease. In the presented review we gathered data from studies conducted on healthy rats where the influence of different nutritional supplements on gut microbiota was assessed. When comparing the observed modulatory effects of tested supplements, one has to keep in mind that scientist used different techniques of microbiota analysis and different strains, gender, and age of rats. A need for standardization of experimental procedures and guidelines that would enhance reproducibility and comparability across microbiome studies in animal models, can be indeed, deduced from the rat studies included in this review as well. No commonly acknowledged standards for the choice of adequate rat strain, gender, sample size, diet, housing environment, or techniques for microbiome analysis are currently in place. All these factors remain therefore possible confounders and vary among different research according to the specific needs of the experiment, availability of resources, and research design. In studies included in this review, microbiota responded very differently to different supplements, but some microbial shifts were seen more frequently and some were common for different groups of supplements. For instance, a change in *Firmicutes/Bacteroidetes* ratio is observed frequently, whereas the increase of *Lactobacillus* is often observed upon prebiotic and probiotic supplementation. The scientists have provided evidence on beneficial effects of different nutritional supplements on the microbiota composition and function in rats that suggests a beneficial role of these supplements in human as well. The modulation of microbiota members whose compositional shifts and functional changes may be an important line of defense against diseases, may be an important research field in the years to come.
